# Inherited Platelet GPIV Deficiency: First Description of a Series of Unrelated Patients with Bleeding Diathesis

**DOI:** 10.3390/biom16081205

**Published:** 2026-08-18

**Authors:** Loredana Bury, Silvia Sorrentino, Emanuela Falcinelli, Giuseppe Guglielmini, Antonietta Ferretti, Paola Concolino, Ana Sánchez-Fuentes, José Rivera, Paolo Gresele, Erica De Candia

**Affiliations:** 1 Section of Internal and Cardiovascular Medicine, Hemostasis and Thrombosis Center, Department of Medicine and Surgery, University of Perugia, 06132 Perugia, Italy; loredana.bury@unipg.it (L.B.); emanuelafalcinelli@gmail.com (E.F.);; 2 Dipartimento di Scienze di Laboratorio ed Ematologiche, Fondazione Policlinico Universitario Agostino Gemelli IRCCS, 00168 Rome, Italy; silvia.sorrentino@policlinicogemelli.it (S.S.); erica.decandia@unicatt.it (E.D.C.); 3 Departmental Unit of Molecular and Genomic Diagnostics, Fondazione Policlinico Universitario Agostino Gemelli IRCCS, 00168 Rome, Italy; 4 Genomics Core Facility, G-STeP, Fondazione Policlinico Universitario Agostino Gemelli IRCCS, 00168 Rome, Italy; 5 Servicio de Hematología, Hospital Universitario Morales Meseguer, Centro Regional de Hemodonación, Universidad de Murcia, IMIB-Pascual Parrilla, CIBERER-ISCIII, 30001 Murcia, Spain; ana.sanchez1610@gmail.com (A.S.-F.);; 6 Grupo Español de Alteraciones Plaquetarias Congénitas (GEAPC)-SETH, 28001 Madrid, Spain; 7 Dipartimento di Medicina e Chirurgia Traslazionale, Facoltà di Medicina, Università Cattolica del Sacro Cuore, 00168 Rome, Italy

**Keywords:** CD36, GPIV, platelets, bleeding

## Abstract

GPIV (CD36) is a multifunctional membrane protein expressed on various cells, including platelets, where it plays a role in adhesion and activation through the interaction with its ligands, including collagen types I and III and thrombospondin 1. Inherited GPIV deficiency, historically recognized in anti-Naka alloimmunized East Asian donors, is considered asymptomatic and associated with normal platelet aggregation, although impaired adhesion under high-flow conditions has been reported. Here, we reconsider the molecular basis, epidemiology and functional consequences of GPIV deficiency and report four unrelated patients in whom heterozygous *CD36* variants are associated with markedly reduced platelet GPIV expression and a clinically relevant mucocutaneous bleeding diathesis. Patients suffered lifelong bleeding symptoms despite normal light-transmission aggregometry and platelet granule content and release and displayed decreased GPIV expression. Three of them showed slightly decreased VWF. Platelet adhesion to Type I collagen was reduced at high shear. These cases suggest for the first time an association between *CD36* gene variants and bleeding and underscore the importance of including GPIV in the diagnostic workup of inherited platelet disorders, particularly when conventional assays do not reveal abnormalities.

## 1. Introduction

Platelet glycoprotein IV (GPIV), also known as CD36, is an 88-kDa integral membrane protein expressed on a broad range of cells, including platelets, monocytes, macrophages, endothelial cells, adipocytes and cardiomyocytes [1]. Originally identified on the platelet surface in the 1970s [2], GPIV was later recognized as a multifunctional receptor involved in lipid metabolism, immune regulation, and vascular biology. In platelets, GPIV functions as a receptor for thrombospondin-1 (TSP-1) and collagen types I and III, mediating adhesive interactions and participating in signal transduction events that regulate platelet activation. Beyond hemostasis, GPIV is a key scavenger receptor responsible for the uptake of oxidized low-density lipoproteins (oxLDL) and long-chain fatty acids, thereby participating in lipid metabolism, inflammation, atherogenesis, and thrombosis [3,4,5].

The *CD36* gene, located on chromosome 7, contains 15 exons, 12 of which are coding [4]. It exhibits complex transcriptional regulation, with multiple promoters and alternative first exons leading to tissue-specific isoforms [6].

Gene variants in *CD36* cause GPIV deficiency, an uncommon but well-characterized inherited condition first described in Japanese blood donors. Two major phenotypes are recognized, Type I and Type II deficiencies, depending on the absence of GPIV on both platelets and monocytes or only on platelets [7].

The molecular basis of these conditions includes missense or nonsense mutations, frameshifts, splice-site defects, and regulatory variants [7].

Although early studies suggested that platelet GPIV acts as a collagen or thrombospondin receptor, its precise contribution to hemostasis remains debated, but it is suggested that GPIV plays a modulatory rather than an essential role in platelet adhesion and activation [8,9,10].

The present article aims to summarize the current knowledge on the molecular basis, epidemiology, and functional consequences of GPIV deficiency, with a particular focus on platelet physiology, and to report a series of unrelated patients with a lifelong mucocutaneous bleeding diathesis in whom heterozygous variants in *CD36* were identified, resulting in markedly reduced platelet GPIV expression. To our knowledge, these are the first documented cases linking inherited GPIV deficiency to a clinically significant bleeding tendency.

## 2. GPIV Structure and Function

GPIV, coded by the *CD36* gene, is an 88-kDa transmembrane glycoprotein composed of two short cytoplasmic tails, two transmembrane domains, and a large extracellular loop that forms a hydrophobic pocket responsible for ligand binding [11]. The extracellular domain contains ten potential N-glycosylation sites and several disulfide bonds that stabilize its tertiary structure. Post-translational modifications—palmitoylation, glycosylation, phosphorylation, and ubiquitination—influence GPIV localization, turnover, and signaling activity [12].

**Figure 1:**
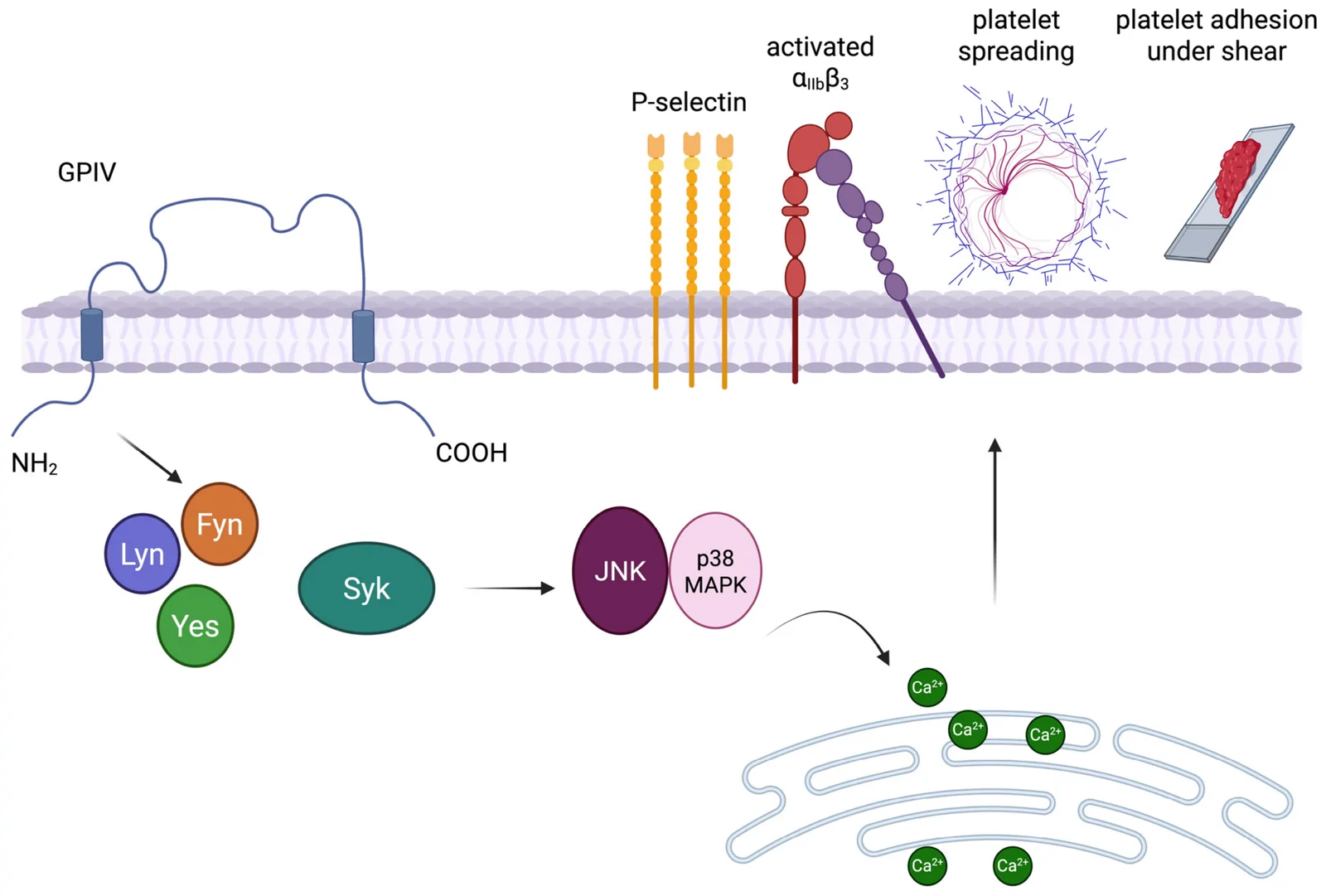
GPIV-mediated signaling and functional platelet responses. Schematic representation of GPIV signaling pathways in platelets and their downstream functional consequences. Engagement of GPIV leads to activation of Src family kinases (Lyn, Fyn, and Yes) and the tyrosine kinase Syk, followed by activation of MAP kinase pathways, including JNK and p38 MAPK. These signaling events promote intracellular calcium mobilization and platelet activation, resulting in surface expression of P-selectin, activation of integrin α_IIb_β_3_, platelet spreading and platelet adhesion under shear conditions.

The N-terminal cytoplasmic region and the C-terminal domain anchor the receptor to the plasma membrane and interact with signaling molecules such as Src-family kinases, enabling downstream activation in platelets and other cells (Figure 1). The central extracellular region, highly glycosylated and cysteine-rich, mediates binding to oxidized phospholipids, fatty acids, thrombospondin-1, and collagen. Distinct motifs within this region are responsible for ligand specificity: hydrophobic stretches accommodate long-chain fatty acids, while surface-exposed loops interact with adhesive proteins [7].

Six isoforms of human GPIV have been described, ranging from 317 to 472 amino acids in length, generated by alternative splicing of the primary transcript [12].

In platelets, GPIV is expressed in 10,000 to 25,000 copies on the cell membrane, the luminal side of the open canalicular system, and the inner surface of α-granules. It acts not only as a receptor for subendothelial ligands such as collagen and thrombospondin-1 but also binds oxidized phospholipids and circulating microparticles released from activated or apoptotic cells [4,13,14]. GPIV is not a classical signaling receptor, as it lacks canonical intracellular signaling motifs. Nonetheless, it engages a well-defined signaling architecture by functioning as a molecular hub in cholesterol-rich membrane microdomains that clusters adaptor proteins and non-receptor tyrosine kinases. Upon ligand binding, GPIV undergoes rapid redistribution into detergent-resistant membrane domains, facilitating the assembly of signaling complexes [7].

Ligand binding initiates signaling via the Src-family kinases Lyn, Fyn and Yes, these in turn propagate signals to Syk, JNK, and p38 MAPK, resulting in increased intracellular Ca^2+^ levels and downstream platelet responses, including platelet spreading, P-selectin expression, α_IIb_β_3_ activation and the potentiation of collagen-triggered thrombus formation, particularly during the early phases of shear-dependent adhesion and aggregation [1,10,15,16,17] (Figure 1) (see also Section 5).

Beyond its role in platelet–collagen interactions under flow, GPIV also mediates adhesive crosstalk between platelets and red blood cells undergoing programmed cell death. Indeed, it was shown that phosphatidylserine-positive erythrocytes adhere to immobilized platelets activated with ADP or thrombin. This adhesion required phosphatidylserine on erythrocytes and GPIV on platelets, since neutralizing antibodies against GPIV markedly reduced erythrocyte adhesion [18]. These findings suggest that the platelet GPIV–erythrocyte phosphatidylserine interaction may contribute to prothrombotic states in conditions associated with increased eryptosis, such as diabetes, renal failure, and hemolytic disorders. Moreover, during a malarial infection, GPIV mediates the binding of platelets to *P. falciparum*–infected red cells; this also causes platelet activation and release of PF4 from platelets. PF4 in turn kills the parasite through the binding to red blood cells via the Duffy antigen [19].

## 3. GPIV Deficiency

Inherited GPIV deficiency is classically divided into two phenotypes: Type I deficiency, defined by the absence of GPIV on both platelets and monocytes/macrophages, and Type II deficiency, in which GPIV is absent only on platelets (see Section 4).

The condition exhibits a striking ethnic distribution: it is rather frequent in East Asian (2.14% in Thailand, 4.13% in China) and African (4.8%) populations but is rare in Europeans and Americans (0.3%) [20,21,22,23,24,25,26]. In Japan, it has been reported that the frequencies of Type I and Type II deficiencies are 0.3–0.5% and 4–5.7%, respectively [20,21].

Most individuals with GPIV deficiency are clinically asymptomatic, and GPIV deficiency has been recognized mainly in transfusion medicine because individuals lacking GPIV (Nak^a^- phenotype) may develop anti-GPIV alloantibodies during pregnancy or after transfusion. Women with Type I deficiency carrying a GPIV-positive fetus may develop anti-GPIV alloantibodies, which cross the placenta and cause fetal and neonatal alloimmune thrombocytopenia (FNAIT). This immune reaction may result in severe thrombocytopenia, hydrops fetalis and intracranial hemorrhage, underscoring the need for prenatal monitoring [22].

Similarly, transfusion of GPIV-positive platelets to a deficient recipient can elicit an alloimmune response with the production of anti-GPIV antibodies causing platelet transfusion refractoriness, a condition characterized by rapid platelet clearance and failure to achieve post-transfusion increments, or post-transfusion purpura [23].

Some authors have suggested that common variants in the *CD36* gene modulate lipid metabolism and cardiovascular risk in Caucasians: in a population of nondiabetic individuals of Caucasian ancestry, a particular haplotype represented by five common SNVs was associated with increased risk of coronary artery disease [24].

*Cd36* knockout (*Cd36*^−/−^) mice display defects in long-chain fatty acid uptake in heart, muscle and adipose tissue, and *Cd36*^−/−^/ApoE^−/−^ double knockout mice show markedly reduced atherosclerotic lesions due to impaired macrophage uptake of oxidized LDL [25]. Moreover, platelets from *Cd36*^−/−^ mice fail to respond to oxidized lipids: they do not accumulate superoxide or hydrogen peroxide, do not activate Src kinases or NOX2, and show impaired phosphorylation of the redox-sensitive kinase ERK5 upon oxLDL exposure. This translates into abolished potentiation by hyperlipidemia of integrin αIIbβ3 activation, P-selectin expression, aggregation, and thrombus formation on collagen under arterial shear rate [26].

Furthermore, *Cd36*^−/−^ mice exhibit a low bone-mass phenotype, characterized by reduced trabecular volume, increased trabecular separation, and decreased trabecular number. These skeletal abnormalities reflect a primary defect in bone formation, as shown by significantly reduced osteoblast perimeter, decreased bone formation rate, and lower circulating markers of bone formation, suggesting that GPIV is required for proper osteoblast differentiation and function [27]. So far, no cases of bone or cartilage alterations in GPIV-deficient patients have been reported.

## 4. Molecular Basis of GPIV Deficiency

**Figure 2:**
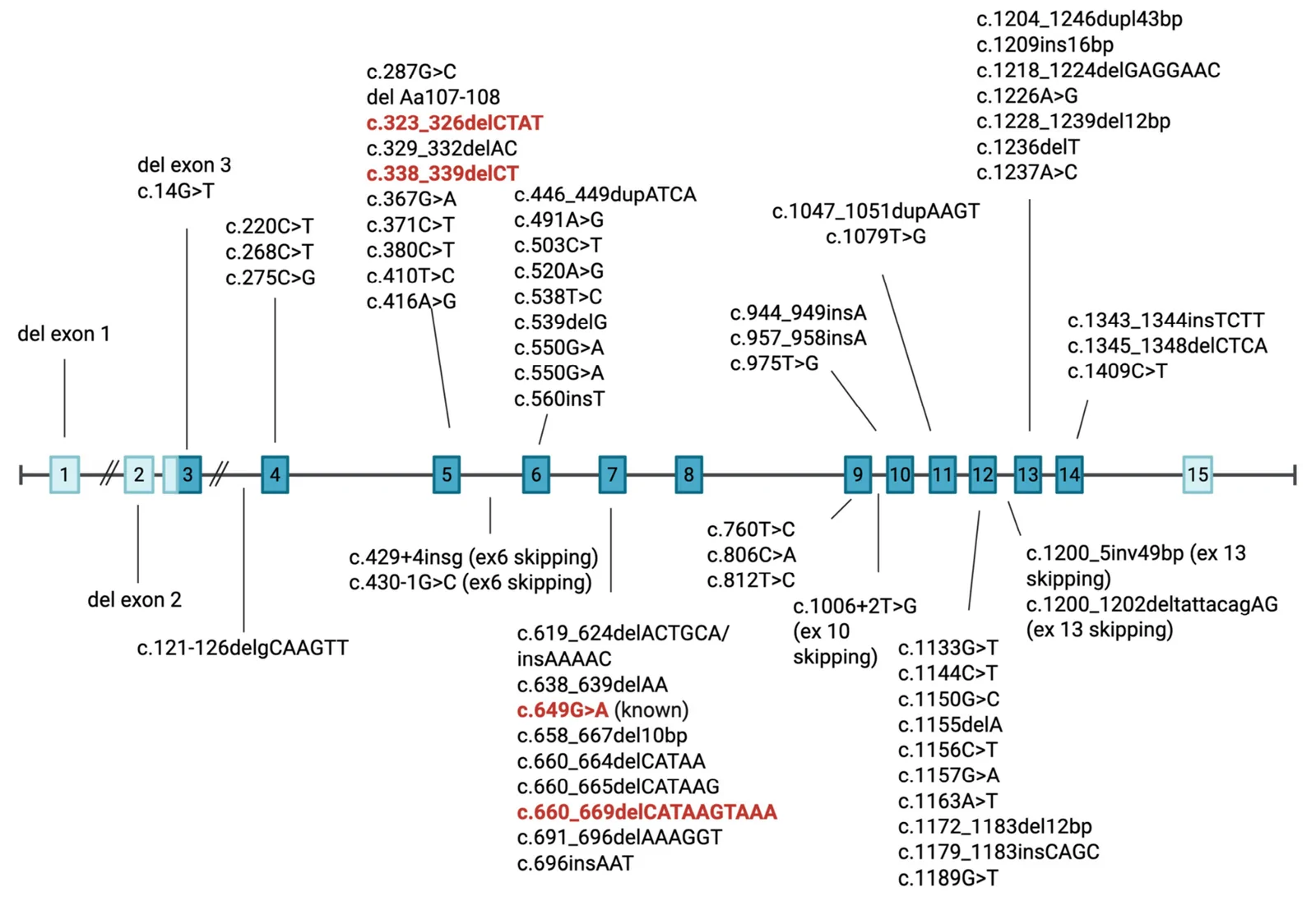
Schematic representation of *CD36* gene structure and reported variants. Diagram of the *CD36* gene showing the exon organization and the location of reported genetic variants associated with GPIV deficiency. Exons are indicated as numbered boxes along the genomic sequence, with variants mapped above or below the gene structure. The variants identified in the patients described in this study are in red; the *CD36* c.649G>A variant identified in one of our patients was previously reported in the literature. The light blue color indicates non coding exons.

The *CD36* gene, located on chromosome 7q11.2, spans approximately 50 kilobases and comprises 15 exons, 12 of which are coding. Exons 1–2 and 15 are noncoding, contributing to the 5′ and 3′ untranslated regions (UTRs), respectively [7] (Figure 2). The gene exhibits marked structural and regulatory complexity, including multiple alternative first exons and promoters that are tissue-specific, allowing fine-tuned expression [7,28,29]. Regulation of *CD36* transcription depends on metabolic and inflammatory stimuli. Transcription factors such as PPARγ, LXRα, and C/EBPα, as well as cytokines including IL-4, TNF-α, and TGF-β, modulate gene expression in a cell-specific manner [7]. In macrophages and adipocytes, for instance, PPARγ activation promotes transcriptional upregulation of GPIV, while phosphorylation of PPARγ through MAPK signaling reduces its activity [30]. Epigenetic mechanisms, including DNA methylation and histone modifications, also contribute to the regulation of GPIV expression under conditions such as oxidative stress and exposure to oxLDL [31].

The *CD36* gene exhibits extensive polymorphism; more than 20 coding mutations have been associated with deficiency. Long-read sequencing of Type I and Type II donors identified 180 gene variants, 12 of which were reported to alter the amino acid sequence. Four of these variants, c.220C>T, c.329_330delAC, c.430-1G>C, and c.1006+2T>G, introduce premature termination codons and produce truncated proteins [32].

The dinucleotide deletion c.329_330delAC (also referred to as 329-330del/AC) is one of the most frequent mutations in East Asian populations [24]. Other recurrent frameshift or nonsense variants include c.1228_1239delATTGTGCCTATT and c.120+1G>T [32].

Type I GPIV deficiency is typically caused by missense or nonsense homozygous or heterozygous mutations; the most frequent are C268T, 949insA, and 329-330delAC or by variants that cause instability of *CD36* mRNA [7].

Type II GPIV deficiency is probably heterogeneous and has been observed in individuals who carry heterozygous mutations (most commonly c.C268T, but also c.329-330 delAC, c.818+108 delAACT, c.T879C, c.1125+13C>A, and c.A1163T) [7,33].

However, some Type II individuals lack detectable coding-region mutations; therefore, some authors proposed that Type II deficiency may result from post-transcriptional mechanisms, such as aberrant tissue-specific pre-mRNA splicing of *CD36* [7].

Genome-wide association studies have identified 23 *CD36* common single-nucleotide variants (SNVs) modulating GPIV platelet surface expression in healthy individuals [34]. Some studies also showed that GPIV expression on platelets is associated with -132A>G of the 5′-untranslated region (UTR) or a silent allele linked to a (TG)n repeat polymorphism in intron 3 [35,36]. The genetic variants in exons and introns that have been associated with GPIV deficiency [37] are shown in Figure 2.

In conclusion, despite progress in identifying genetic variants, the molecular basis of GPIV deficiency remains incompletely understood. Type I deficiency clearly results from complete loss of GPIV expression due to biallelic nonsense, frameshift, or splicing mutations; Type II deficiency, however, encompasses heterozygous mutations, regulatory changes, and unknown post-transcriptional mechanisms, with some individuals lacking any detectable mutation.

Further studies are required to elucidate these mechanisms and to enable precise molecular diagnosis of GPIV deficiency.

## 5. Function of Platelets Lacking GPIV

Despite its role in platelet activation, GPIV deficiency has not been associated with bleeding or major platelet dysfunction. A study carried out on seven healthy Japanese blood donors with GPIV deficiency and no bleeding reported normal responses to collagen, ADP, epinephrine, arachidonic acid and thrombin at light-transmission aggregometry [8]. Another study that investigated platelet function in two Japanese subjects with the Nak^a^- phenotype showed normal or even increased aggregation in response to Type I collagen and normal adhesion to types I, III, and IV collagens [9]. Moreover, blocking α_2_β_1_ integrin with monoclonal antibody 176D7 inhibited aggregation and adhesion to the same extent in Nak^a^- and in normal platelets.

TSP-1 expression after thrombin activation was equivalent in Nak^a^- and normal platelets [9]. These findings show that GPIV is not essential for platelet aggregation in low-shear stress conditions or for adhesion to collagen under static conditions.

In contrast, when platelets encounter collagen under flow, GPIV plays a modulatory role in the early tethering. In a perfusion system at 800 s^−1^ shear, Diaz-Ricart et al. demonstrated that normal platelets incubated with the Fab fragments of an anti-GPIV antibody as well as platelets from a Nak^a-^ donor displayed reduced surface coverage, especially at early time points. Although overall adhesion finally reached near-normal levels, the delay in the initial attachment phase suggests that GPIV accelerates initial adhesion, but it becomes redundant once stable contacts form [10].

Subsequent experiments showed that when normal blood treated with polyclonal anti-GPIV Fab or monoclonal antibodies against GPIV was perfused over rabbit aorta segments or endothelial cell extracellular matrix platelet adhesion was reduced by about 30–40% at early time points. Blood from a Nak^a^- donor yielded comparable inhibition [16]. These data confirmed that platelet GPIV mediates early interactions with subendothelial collagen and extracellular matrix, particularly at high shear, but is less important for sustained adhesion.

The absence of overt hemostatic defects in GPIV-deficient individuals suggests that compensatory adhesive pathways, mediated by other collagen receptors (e.g., GPVI and integrin α2β1), can maintain effective primary hemostasis.

## 6. Description of Four Cases with GPIV Deficiency and a Bleeding Diathesis

Here we report four unrelated individuals with a clinically relevant bleeding phenotype who were found to carry heterozygous variants in *CD36* leading to a marked reduction in platelet GPIV surface expression and who represent the first reported cases in which an inherited GPIV deficiency is associated with a hemorrhagic diathesis.

All studies were approved by the responsible institutional review boards (Ethics Board of the University of Perugia n.13/2024; Ethics Committee of the Reina Sofía University Hospital of Murcia, Spain; last approval 28 November 2023) and/or followed the locally approved rules for the use of data and biological samples (mod. 341, version 1, 6 July 2022 Fondazione Policlinico Universitario A. Gemelli IRCCS) and were carried out in conformity with the Declaration of Helsinki.

*Case 1.* The first patient (P1) is a 22-year-old woman with easy bruising, bleeding from minor wounds, prolonged bleeding following dental extractions, and an ISTH-BAT bleeding score (BS) of 5, which is the upper normal limit for women [38]. She had previously received a clinical diagnosis of Ehlers–Danlos syndrome due to joint hypermobility, although not confirmed by genetic testing. No family history of bleeding was reported.

*Case 2.* The second patient (P2) is a 50-year-old woman who presented with a history of easy bruising and spontaneous petechiae, nose and gum bleeding, abnormal uterine bleeding (AUB), prolonged bleeding following dental extractions (n = 3), spontaneous bleeding into a corpus luteum requiring access to the emergency department at the age of 43, cutaneous purpura after delivery at the age of 29, and two episodes of rectal bleeding without rectal lesions at the ages of 42 and 49 and had an ISTH-BAT BS of 10.

In addition, P2 presented a spectrum of bone-related abnormalities, including osteolipomas, one of which, located in the iliopsoas muscle, required surgical removal with associated profuse bleeding; and right ear otosclerosis, which required a surgical procedure with associated intra-operative profuse bleeding. A subsequent surgical procedure for left ear otosclerosis performed after desmopressin and tranexamic acid administration was not complicated by bleeding. Due to the finding of an atrial septal defect during investigations for episodes of amaurosis fugax, she underwent percutaneous closure with a MemoPart device after the administration of desmopressin, without bleeding complications. After this procedure, the patient was started on aspirin. Thus, aspirin therapy was initiated after the onset of the bleeding manifestations but this does not explain the patient’s clinical phenotype.

Two male siblings suffered from nosebleed episodes during childhood, and their mother experienced excessive bleeding requiring red blood cell transfusion in one out of three deliveries, while the father did not report bleeding symptoms.

*Case 3*

The third patient (P3) is a 62-year-old woman with a previous diagnosis of mild von Willebrand disease (VWD). She reported a lifelong history of mucocutaneous bleeding, including easy bruising, recurrent spontaneous epistaxis, and gingival bleeding. She also experienced prolonged bleeding after multiple dental extractions, and her ISTH-BAT BS was 9. Subsequently, she underwent surgery for Reinke’s edema and additional dental extractions under hemostatic replacement therapy, without significant bleeding complications. She also had three uncomplicated vaginal deliveries, with no bleeding complications during delivery or in the postpartum period. Her mother also reported spontaneous bruising and gingival bleeding, suggesting an inherited bleeding tendency. She reported suffering from arthritis.

*Case 4*

The fourth patient (P4) is a 35-year-old woman who was referred for investigation of a bleeding diathesis after developing a disproportionately large facial hematoma following local anesthesia for a dental procedure. She also reported heavy menstrual bleeding leading to iron deficiency, that required oral iron supplementation, and delayed cessation of bleeding after minor cuts. Her ISTH-BAT BS was 6. Her father had a history of recurrent nosebleeds.

### Laboratory Findings and Molecular Analysis

Given the patients’ mucocutaneous bleeding symptoms and abnormal/upper normal limit ISTH-BAT BS, we proceeded with an in-depth evaluation of platelet function [39,40]. At the time of platelet function testing, patients confirmed that they had not taken drugs possibly interfering with platelet function.

In P1, routine blood clotting assays [41] were normal. Von Willebrand factor (VWF) values were at the lower limit of normality or slightly reduced for blood group A (VWF:Ag 54%, normal value (n.v.) 61–152%; VWF:RCo 50%, n.v. 58–175%; VWF:CBA 61%, n.v. 50–400%). FVIII was normal. Platelet count was normal (155 ± 5 × 10^6^ platelets/mL) with a normal mean platelet volume (MPV) (10 fL; controls: 9.87 ± 1.2 fL). The PFA-100^®^ (Dade-Behring, Deerfield, IL, USA) showed prolonged closure times with both collagen/ADP (C/ADP) and collagen/epinephrine (C/Epi) cartridges (C/ADP 140 s, n.v. 62–100 s; C/Epi 185 s, n.v. 82–150 s), suggesting a possible defect in primary hemostasis.

In P2, VWF was moderately reduced or in the low range on two different occasions (VWF antigen 57% and 65%, respectively, n.v. 61–152%; VWF:RCo 48% and 61%, respectively, n.v. 58–175%); FVIII and FXIII were normal. Platelet count was normal (170 × 10^6^ platelets/mL) with a normal mean platelet volume (MPV) (10.3 fL; controls: 9.87 ± 1.2 fL). The skin bleeding time was prolonged (14 min, n.v. 2–9 min), suggesting a possible defect in primary hemostasis. PFA-100 closure time was within normal range (Coll/EPI = 109 s, n.v. < 140 s, Coll/ADP = 97 s, n.v. < 100 s).

In P3, the platelet count was 175 × 10^9^/L with a slightly increased mean platelet volume (12.8 fL; controls: 8.8–12.1); the VWF antigen was in the low range (61%), and VWF:RCo slightly decreased (49%); FVIII was normal. The PFA-100 closure times were prolonged (C/Epi 181 s, n.v. 76–131 s; C/ADP 137 s, n.v. 57–100 s).

In P4, the platelet count was 271 × 10^9^/L with normal platelet volume (11 fL; controls: 8.8–12.1). She also had an isolated prolongation of the PFA-100 C/Epi closure time (218 s, n.v. 76–131 s), which was confirmed on repeat analysis. VWF antigen and RCo were normal (119% and 114%, respectively). Because PFA-100 closure time is influenced by multiple determinants, including VWF levels, it cannot be excluded that the borderline-low VWF levels detected in three of our patients may have contributed to the prolonged closure times observed.

In all patients, light transmission aggregometry was normal with all tested agonists, including ADP, epinephrine, collagen Type I, convulxin (CVX), collagen-related peptide (CRP), arachidonic acid, U46619 and TRAP-6 [42,43,44], except for a slight reduction in the response to CVX in P1 (Supplementary Table S1).

α-granule content was normal, as shown by normal levels of β-thromboglobulin in resting platelet lysates as assessed by ELISA (Asserachrom^®^ β-TG Diagnostica Stago, Paris, France) [39,45] in P1. Also α-granule release was normal, as assessed by β-TG release in the supernatant [39,45] in P1 and by platelet surface P-selectin expression [42] in P1, P2, P3 and P4 [42,43], except for P3, which showed an impaired platelet P-selectin expression in response to CRP (Supplementary Table S1).

Similarly, δ-granule content and secretion were essentially normal, as shown by mepacrine uptake and release [42] in P1 and P2, by ATP release measured by lumiaggregometry in P1 [38], and by GPIV expression by flow cytometry in P3 and P4 [46]. Interestingly, P3 also showed an impaired platelet CD63 expression in response to CRP (Supplementary Table S1). Altogether, these findings excluded δ- or α-granule-relevant disorders.

α_IIb_β_3_ activation assessed by PAC-1 binding by flow cytometry [47] in P1 and by fluorescent fibrinogen binding [44] in P3 and P4 was also normal (Supplementary Table S1), except for P3, which, again, showed impaired fibrinogen binding in response to CRP.

**Figure 3:**
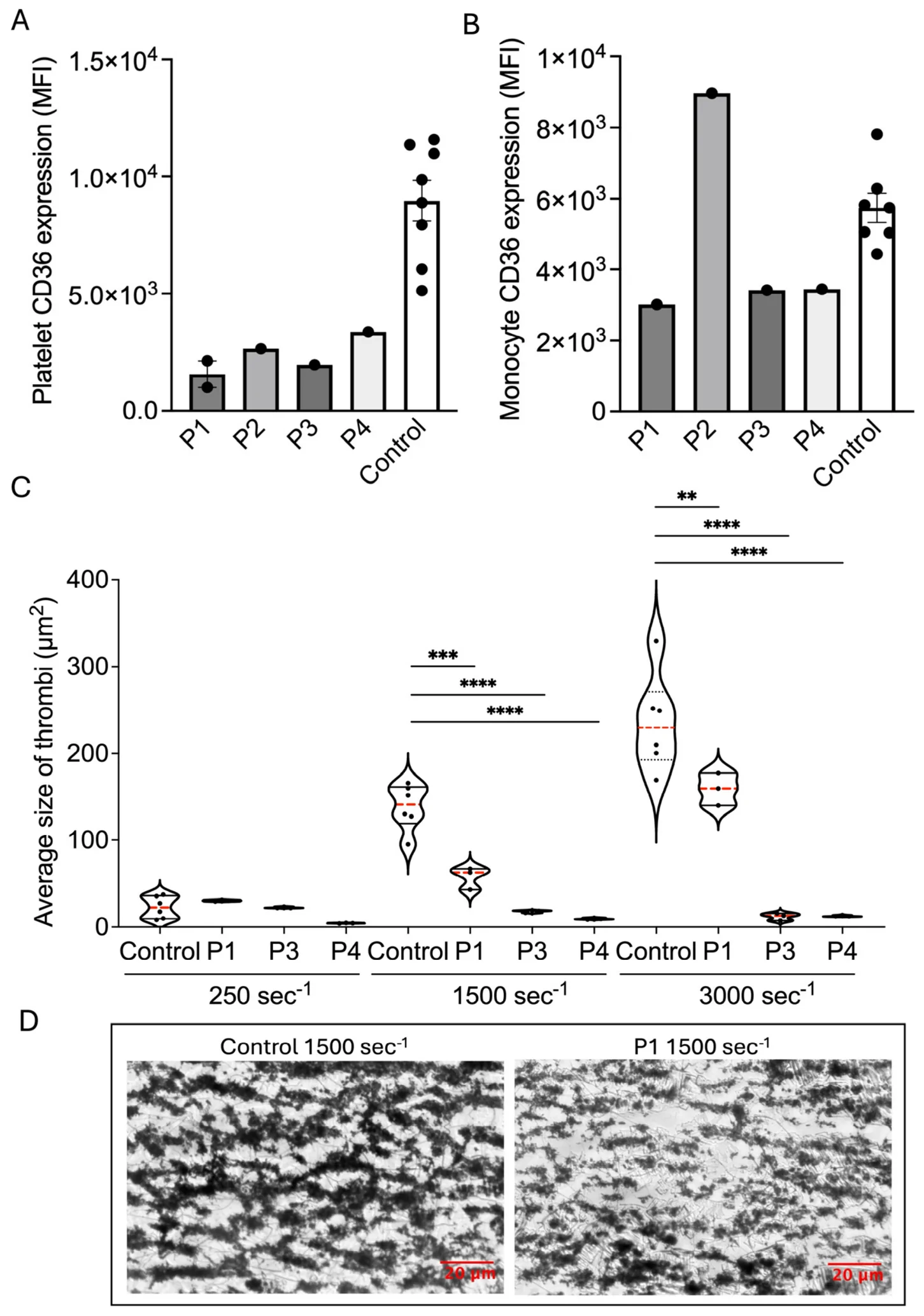
Reduced GPIV expression and impaired thrombus formation under flow in GPIV-deficient patients. (**A**) Platelet GPIV surface expression measured by flow cytometry in patients and healthy controls, reported as median fluorescence intensity (MFI). Patients show markedly reduced platelet GPIV expression compared with controls. (**B**) Monocyte GPIV surface expression assessed by flow cytometry in patients and healthy controls, reported as median fluorescence intensity (MFI). Reduced GPIV expression is observed in P1, P3 and P4 monocytes compared with controls. (**C**) Quantification of thrombus formation on collagen under flow conditions at increasing shear rates (250, 1500, and 3000 s^−1^). The average thrombus size (µm^2^) is shown for controls, P1, P3 and P4. Thrombus formation is markedly reduced in the patient, particularly at higher shear rates. Each dot represents a replicate (technical replicates for patients, biological replicates for controls); violin plots illustrate the distribution of thrombus sizes with the median indicated by a dashed red line. ** *p* < 0.01; *** *p* < 0.001; **** *p* < 0.0001, two-way ANOVA. (**D**) Representative images of thrombi formed under flow at 1500 s^−1^ in control and P1.

The only consistent abnormality was detected by flow cytometry of platelet surface glycoproteins [42]. While all major receptors, including GPIb/IX/V, α_2_β_1_, GPVI, and α_IIb_β_3_, were expressed at normal levels (Supplementary Table S1), GPIV expression was markedly reduced in all patients, reaching only about 10% of normal in P1 and P3 and 15–20% in P2 and P4, confirming a selective defect of this receptor (Figure 3A). Moreover, GPIV expression in monocytes was decreased in P1, P3 and P4 (about 55% of normal) and normal in P2 (Figure 3B). This suggests Type I GPIV deficiency in P1, P3 and P4 and Type II in P2. All patients showed partial GPIV deficiency, characterized by markedly reduced rather than absent platelet GPIV expression. They were classified as Type I or Type II based on the distribution of GPIV expression on platelets and monocytes, respectively [7].

Platelet adhesion on a Type I collagen-coated surface under flow conditions [48,49] was assessed for P1, P3 and P4, while this was not possible for P2 due to aspirin intake.

At low shear rates (250 s^−1^), when adhesion to collagen is highly dependent on the GPIbα interaction with VWF [50], platelet adhesion and thrombus formation were comparable to controls, while at high shear rates (1500 and 3000 s^−1^), when the collagen receptors have a role in adhesion [51,52], platelets formed smaller thrombi, compatible with a collagen receptor defect (Figure 3C,D).

Given these results, clinical exome sequencing was performed in P1 and P2, upon patients’ written informed consent, on DNA extracted from peripheral venous blood samples using the capture-based NGS Clinical Exome Solution^®^ v3 kit (SOPHiA Genetics, Rolle, Switzerland) that covers the coding regions and splicing junctions of 6380 genes related to rare and inherited conditions. Sequencing was carried out on the NovaSeq 6000 System NGS platform (Illumina, San Diego, CA, USA). Tertiary analysis was performed by the Sophia DDM^®^ v4 platform (Sophia Genetics) to detect SNVs, indels, and Copy Number Variations (CNVs) [53]. Firstly, we designed a virtual panel of 94 genes associated with bleeding, thrombotic, and platelet disorders according to the guidelines of the International Society on Thrombosis and Haemostasis (ISTH) [54]. Since this first screening was silent, we extended the analysis to the whole exome, identifying two heterozygous frameshift variants in the *CD36* gene: *CD36* (NM_001001548.3):c.660_669del (p.Asn220Lysfs*4) in P1 and *CD36* (NM_001001548.3):c.338_339del (p.Ser113Phefs*20) in P2 (Figure 2).

For P3 and P4, genetic analysis was carried out by an expanded high-throughput sequencing (HTS) gene panel [46,55] and identified the heterozygous *CD36* (NM_001001548.3):c.323_326del (p.Glu108AlafsTer48) variant in P3 and the heterozygous *CD36* (NM_001001548.3):c.649G>A (p.Gly217Arg) variant in P4 (Supplementary Table S2).

The *CD36* (NM_001001548.3):c.660_669del variant, found in P1, is reported in the ClinVar database (Variation ID: 810109) with conflicting classifications. We classified it as likely pathogenic according to the recommendation of the ACMG/AMP guideline [56]. It has indeed a very low frequency (0.00003285) in gnomAD and is predicted to result in a loss or disruption of normal protein function through nonsense-mediated decay (NMD) or protein truncation. The strikingly reduced expression of GPIV on the platelet and monocyte surface further supports its pathogenicity. The variant has been previously reported to be associated with *CD36*-related disorder [57].

To our knowledge, the second variant, *CD36* (NM_001001548.3):c.338_339del, found in P2, has not been reported in the literature, but it is listed in the ClinVar database (Variation ID: 1302901) as a Variant of Uncertain Significance (VUS). However, both the in silico predictive models (https://varsome.com) and the ACMG guidelines [56] agree in classifying this variant as likely pathogenic. In fact, it is predicted to result in a frameshift and premature protein termination (p.Ser113Phefs*20) with the elimination of important functional domains; it is present in gnomAD at a very low frequency (0.00004028). Moreover, several pathogenic variants have been found near this variant [7,32,33].

The third variant, *CD36* (NM_001001548.3):c.323_326del, found in P3, has not been reported previously and is absent from gnomAD and ClinVar. According to ACMG guidelines, this variant is likely pathogenic because it is predicted to result in a frameshift and premature protein termination (p.Glu108AlafsTer48) and is absent from gnomAD.

The fourth variant, CD36 (NM_001001548.3):c.649G>A, found in P4, is present in ClinVar (variation ID 360761) with conflicting classifications of pathogenicity (four VUS and one likely benign); moreover, it was reported in individuals with GPIV deficiency in the homozygous, compound heterozygous and heterozygous states [58]. It is present in gnomAD at a very low frequency (0.0002), and in silico prediction tools consistently support a deleterious effect of the variant. We classified this variant as VUS.

## 7. Discussion

Until now, inherited GPIV/CD36 deficiency has been considered an essentially asymptomatic gene variant with reduced platelet GPIV expression but no consistent association with bleeding symptoms or platelet dysfunction.

The cases described here expand the clinical spectrum of inherited GPIV deficiency, suggesting that, although this condition is generally asymptomatic, in rare instances it may be associated with a mild-to-moderate bleeding tendency. All patients displayed markedly reduced platelet GPIV expression. The degree of residual GPIV expression observed in our heterozygous patients is lower than expected based on haploinsufficiency alone. Previous studies have shown marked interindividual variability in platelet GPIV expression, suggesting that regulatory variants or other heritable platelet-specific factors may modulate the expression of the wild-type allele [34]. Consistent with this, we also observed substantial variability in platelet GPIV expression among the healthy controls in our study. We speculate that the heterozygous loss-of-function variants identified in our patients act in the context of additional genetic and/or regulatory factors, resulting in GPIV levels substantially lower than the approximately 50% that would be predicted from simple haploinsufficiency. For instance, GPIV undergoes multiple post-translational modifications, including ubiquitination, glycosylation, palmitoylation and phosphorylation, which can regulate its stability, trafficking and functional activity [59]. Although these mechanisms have not been investigated in our patients, they may represent additional determinants of interindividual variability in platelet GPIV expression.

Functional studies demonstrated, overall, normal aggregation and secretion responses to conventional agonists, indicating that GPIV is not required for platelet activation under static or low shear stress conditions. However, the prolonged closure times at PFA-100^®^, prolonged bleeding time [60], and the patients’ mucocutaneous bleeding symptoms point to a defect in platelet adhesion under high shear stress, which may impact the initial phases of primary hemostasis, consistent with previous observations in Nak^a^- individuals [8,9,10] and with in vitro data showing that GPIV facilitates the early stages of platelet–collagen interaction in flow conditions [16]. These findings raise the possibility that even a mild impairment of GPIV-dependent adhesion to collagen may become clinically relevant, especially in individuals with additional factors affecting collagen integrity or vascular connective tissue. Notably, two of our patients displayed additional clinical features: P1 presented joint hypermobility with a previous clinical diagnosis of Ehlers–Danlos syndrome, suggestive of an underlying collagen-related fragility, whereas P2 exhibited distinct bone-associated abnormalities, including an osteolipoma, a very rare histological variant of lipoma, and bilateral otosclerosis. Notably, no pathogenic variants were identified in *VWF* or Ehlers–Danlos syndrome-related genes.

The skeletal findings observed in some of our patients prompted us to consider whether they might be related to GPIV function. Studies in *Cd36*^−/−^ mice have shown that GPIV contributes to osteoblast differentiation and bone matrix formation [27], and altered GPIV expression has been reported in patients with ameloblastoma, a benign tumor of the maxillary bones [61]. However, any association between congenital GPIV deficiency and skeletal manifestations in humans remains speculative and will require confirmation by the observation of additional patients and by functional studies.

Finally, we observed borderline-low VWF levels in three of our four patients with GPIV deficiency. Although this finding may simply reflect biological variation, it raises the possibility of a systematic association. Indeed, Pancratz and colleagues showed in genome-wide studies that a nonsense variant (p. Tyr325Ter) in *CD36* is significantly associated with FVIII levels and also showed a trend to association with VWF levels (*p* = 0.0037) [62]. Moreover, de Vries et al. showed that CD36 silencing reduced VWF release from HUVECs [63]. However, our data do not allow any conclusions regarding the underlying mechanism, and specific studies, including VWF multimer analysis, VWF propeptide measurements and endothelial functional assays, will be required to determine whether GPIV deficiency has any effect on VWF biology.

Taken together, our findings support the concept that GPIV acts as an accessory receptor that modulates platelet adhesion and activation in high-shear-rate vascular beds. Genetic heterogeneity and variable GPIV expression levels might account for the wide spectrum of bleeding phenotypes observed in GPIV deficiency. It cannot be excluded that other genetic and/or environmental factors might contribute to this clinical variability. One possible hypothesis is that the cumulative effect of common genetic variants influencing platelet count or function may modify the clinical expression of GPIV deficiency. Recent data from large population cohorts have shown that common variants with small individual effects can, when combined into a polygenic score (PGS), substantially modify the penetrance and expressivity of rare pathogenic variants in genes implicated in bleeding and platelet disorders [64]. Although no PGS analysis was performed in the present study, future investigations could explore whether polygenic background contributes to the variability of the bleeding phenotype in GPIV deficiency. Additional contributors may include epigenetic regulation, co-inheritance of variants in extracellular matrix or collagen-related genes, or acquired factors such as inflammation or hormonal status. In this regard, recent single-cell transcriptomic studies have shown that aging and sex can substantially remodel megakaryocyte subpopulations and transcriptional programs, for instance, those involved in platelet activation and receptor expression [65], supporting the concept that the platelet phenotype may be influenced by biological modifiers beyond the underlying genetic defect.

The bleeding phenotype observed in our patients should be interpreted with caution, as it may be multifactorial in at least some of the cases. Nevertheless, the consistent platelet functional abnormalities observed in our cohort, together with previous experimental and clinical evidence supporting a role for GPIV in platelet activation and thrombus formation, support the hypothesis that GPIV deficiency contributes to the bleeding phenotype.

Recognition of this condition may be clinically relevant not only for its potential impact on hemostasis but also for its immunohematologic implications, such as the risk of anti-GPIV alloimmunization and transfusion refractoriness. Future studies integrating functional platelet assays under flow and molecular analyses in larger cohorts will be required to clarify the contribution of GPIV deficiency to the bleeding phenotype and to better define the diagnostic and therapeutic implications of this rare disorder.

Beyond its role in platelet biology, recent studies have identified CD36 expression as an independent immunophenotypic marker of specific high-risk subtypes of acute leukemia, highlighting its potential value for disease classification and risk stratification [66,67]. Whether these observations have any relevance to inherited GPIV deficiency deserves further investigation.

## 8. Conclusions

In conclusion, our findings expand the clinical spectrum of inherited GPIV deficiency, suggesting that this condition, although generally asymptomatic, may in rare cases contribute to a mild-to-moderate bleeding phenotype, potentially through impaired platelet adhesion under high shear stress and in combination with additional genetic or clinical modifiers.

## Data Availability

The raw data supporting the conclusions of this article will be made available by the authors on request.

## Supplementary Materials

The following supporting information can be downloaded at: https://www.mdpi.com/article/10.3390/biom16081205/s1, Table S1: Platelet function studies; Table S2: ACMG classification of the identified CD36 variants.
