# Supplementary material for: Inherited Platelet GPIV Deficiency: First Description of a Series of Unrelated Patients with Bleeding Diathesis
Source: Biomolecules. 2026 Aug 18;16(8):1205. doi: 10.3390/biom16081205 (PMC13510369; doi:10.3390/biom16081205)
Supplement: Supplementary file 1 [file biomolecules-16-01205-s001.zip › biomolecules-4487730-supplementary.pdf]

Table S1. Platelet function studies.

**Patient 1 (P1)**			**Patient 2 (P2)**			**Patient 3 (P3)**			**Patient 4 (P4)**		
**PFA-100®**	**Result (sec)**	**Normal range**	**PFA-100®**	**Result (sec)**	**Normal range**	**PFA-100®**	**Result (sec)**	**Normal range**	**PFA-100®**	**Result (sec)**	**Normal range**
Coll/EPI	185	82-150	Coll/EPI	109	<140	Coll/EPI	181	76-131	Coll/EPI	**218**	76-131
Coll/ADP	140	62-100	Coll/ADP	97	<100	Coll/ADP	137	57-100	Coll/ADP	89	57-100
**VWF**	**Result (%)**	**Normal range**	**VWF**	**Result (%)**	**Normal range**	**VWF**	**Result (%)**	**Normal range**	**VWF**	**Result (%)**	**Normal range**
VWF:Ag	54	61-152%	VWF:Ag	57-65	61-152%	VWF:Ag	61	61-152%	VWF:Ag	119	61-152%
VWF:RCo	50	58-175%	VWF:RCo	48-61	58-175%	VWF:RCo	49	58-175%	VWF:RCo	114	58-175%
**Platelet aggregation**	**Result (% of maximal aggregation)**	**Normal range**	**Platelet aggregation**	**Result (% of maximal aggregation)**	**Normal range**	**Platelet aggregation**	**Result (% of maximal aggregation)**	**Normal range (N≥10)**	**Platelet aggregation**	**Result (% of maximal aggregation)**	**Normal range (N≥10)**
ADP 5 μM	82.4	41.4-83.4	ADP 4 μM	86	>70	ADP 5 μM	78	68 ± 17	ADP 5 μM	77	68 ± 17
Epinephrine 10 μM	49.4	44.1-69.7	Epinephrine 5 μM	92	>70	Epinephrine	N/A	N/A	Epinephrine	N/A	N/A
Collagen 2 μg/ml	58.4	57.8-80.2	Collagen 2 μg/ml	82	>70	Collagen 2 μg/ml	82	76±17	Collagen 5 μg/ml	95	79 ± 18
Convulxin 10 ng/ml	47.3	51.4-84.8	Convulxin 10 ng/ml	N/A	N/A	CRP 2 μM	93	79±8	CRP 2 μM	N/A	79±8
Arachidonic acid 1mM	80.2	58.1-80.3	Arachidonic acid 1mM	86	>70	Arachidonic acid 1.5mM	89	82 ± 11	Arachidonic acid 1.5 mM	100	82 ± 11
U46619 1μM	83.6	55.6-86.8	U46619 1μM	N/A	N/A	U46619 5μM	86.5	77 ± 13	U46619 5μM	N/A	77 ± 13
TRAP-6 10 μM	78.9	62-82.6	TRAP-6 10 μM	82	>70	TRAP-6 25 μM	87	85 ± 8	TRAP-6 25 μM	81	85 ± 8
RIPA (mg/ml of ristocetin)	0.9	0.7-1	RIPA (0.6 mg/ml of ristocetin)	2	<9	RIPA (1.25 mg/ml of ristocetin)	81	86 ± 9	RIPA (0.8 mg/ml of ristocetin)	N/A	86 ± 9
			RIPA (1.2 mg/ml of ristocetin)	91	>70						

**Flow cytometry**	**Result (MFI)**	**Normal range**	**Flow cytometry**	**Result (MFI)**	**Normal range**	**Flow cytometry**	**Result (MFI)**	**Normal range (N≥100)**	**Flow cytometry**	**Result (MFI)**	**Normal range (N≥100)**
CD42a (GPIX)	34795	31149 – 40826	CD42a (GPIX)	N/A	N/A	CD42a (GPIX)	14479	17578 ±6764	CD42a (GPIX)	29331	19837 ± 6454
CD42b (GPIba)	7066	6294 – 10217	CD42b (GPIba)	N/A	N/A	CD42b (GPIba)	17343	15647 ± 3935	CD42b (GPIba)	18559	15270 ± 2599
CD41 (GPIIb)	39306	36190 – 61133	CD41 (GPIIb)	N/A	N/A	CD41 (GPIIb)	79923	58245 ±15374	CD41 (GPIIb)	45777	42897 ± 17336
CD61 (GPIIIa)	16431	15674 – 27229	CD61 (GPIIIa)	N/A	N/A	CD61 (GPIIIa)	49772	39339 ±7859	CD61 (GPIIIa)	41954	34123 ± 7136
GPVI	N/A	N/A	GPVI	N/A	N/A	GPVI	4081	3877 ±919	GPVI	4642	3681 ± 845
**P-Selectin expression**	**Result (% of positive platelets)**	**Normal range**	**P-Selectin expression**	**Result (% of positive platelets)**	**Normal range**	**P-Selectin expression**	**Result (MFI)**	**Normal range (N≥100)**	**P-Selectin expression**	**Result (MFI)**	**Normal range (N≥100)**
Resting	3.48	4.05-7.04	N/A	N/A	N/A	Resting	181	273 ± 70	Resting	248	273 ± 70
ADP 10 μM	52.11	46.8-73.9	N/A	N/A	N/A	ADP 10 μM	820	1395 ± 697	ADP 10 μM	1333	1395 ± 697
TRAP-6 20 μM	84.76	83.8-93.5	N/A	N/A	N/A	TRAP-6 25 μM	5094	4789 ± 2033	TRAP-6 25 μM	7911	4789 ± 2033
N/A	N/A	N/A	N/A	N/A	N/A	CRP 10μg/mL	1678	6726±2683	N/A	N/A	6726±2683
**β-thromboglo-bulin**	**Result (ng x 10^8 platelets)**	**Normal range**	**β-thromboglobulin**	**Result (ng x 10^8 platelets)**	**Normal range**	**β-thromboglobulin**	**Result (ng x 10^8 platelets)**	**Normal range**	**β-thromboglobulin**	**Result (ng x 10^8 platelets)**	**Normal range**
Content	4418	2940-4527	Content	N/A	N/A	Content	N/A	N/A	Content	N/A	N/A
ADP 5 μM	2771	2013-2854	ADP 5 μM	N/A	N/A	ADP 5 μM	N/A	N/A	ADP 5 μM	N/A	N/A
Epinephrine 10 μM	3377	2310-3860	Epinephrine 10 μM	N/A	N/A	Epinephrine 10 μM	N/A	N/A	Epinephrine 10 μM	N/A	N/A
Collagen 2 μg/ml	2680	2590-3202	Collagen 2 μg/ml	N/A	N/A	Collagen 2 μg/ml	N/A	N/A	Collagen 2 μg/ml	N/A	N/A
Arachidonic acid 1mM	2892	2276-2792	Arachidonic acid 1mM	N/A	N/A	Arachidonic acid 1mM	N/A	N/A	Arachidonic acid 1mM	N/A	N/A

U46619 1μM	2652	1911-2751	U46619 1μM	N/A	N/A	U46619 1μM	N/A	N/A	U46619 1μM	N/A	N/A
TRAP-6 10 μM	2979	2263-2942	TRAP-6 10 μM	N/A	N/A	TRAP-6 10 μM	N/A	N/A	TRAP-6 10 μM	N/A	N/A
**Mepacrine content and release**	**Result (% of positive platelets)**	**Normal range**	**Mepacrine content and release**	**Result (% of positive platelets)**	**Normal range**	**Mepacrine content and release**	**Result (% of positive platelets)**	**Normal range**	**Mepacrine content and release**	**Result (% of positive platelets)**	**Normal range**
Resting	66.4	66.4-77.2	Resting	N/A	N/A	Resting	N/A	N/A	Resting	N/A	N/A
Thrombin 0.05 U/ml	13.8	9.7-18.6	Thrombin 0.05 U/ml	N/A	N/A	Thrombin 0.05 U/ml	N/A	N/A	Thrombin 0.05 U/ml	N/A	N/A

**CD63 expression**	**Result (% of positive platelets)**	**Normal range**	**CD-63 expression**	**Result (% of positive platelets)**	**Normal range**	**CD-63 expression**	**Result (% of positive platelets)**	**Normal range (N≥100)**	**CD-63 expression**	**Result (% of positive platelets)**	**Normal range**
N/A	N/A	N/A	N/A	N/A	N/A	Resting	539	510 ±123	Resting	440	510 ±123
N/A	N/A	N/A	N/A	N/A	N/A	CRP 10 μg/ml	1502	4059 ± 1341	CRP 10 μg/ml	3181	4059 ± 1341
N/A	N/A	N/A	N/A	N/A	N/A	TRAP-6 25 μM	3122	3268 ± 1433	TRAP-6 25 μM	2722	3268 ± 1433
**ATP (lumiaggregometry)**	**Result (ng x 10^8 platelets)**	**Normal range**	**ATP (lumiaggregometry)**	**Result (ng x 10^8 platelets)**	**Normal range**	**ATP (lumiaggregometry)**	**Result (ng x 10^8 platelets)**	**Normal range**	**ATP (lumiaggregometry)**	**Result (ng x 10^8 platelets)**	**Normal range**
ATP content	2.48	1.82-2.94	ATP content	N/A	N/A	N/A	N/A	N/A	N/A	N/A	N/A
ADP 5 μM	0.56	0.39-0.86	ADP 5 μM	N/A	N/A	N/A	N/A	N/A	N/A	N/A	N/A
Epinephrine 10 μM	0.41	0.36-0.92	Epinephrine 10 μM	N/A	N/A	N/A	N/A	N/A	N/A	N/A	N/A
Collagen 2 μg/ml	0.90	0.57-1.2	Collagen 2 μg/ml	N/A	N/A	N/A	N/A	N/A	N/A	N/A	N/A
Arachidonic acid 1mM	0.53	0.45-1.3	Arachidonic acid 1mM	N/A	N/A	N/A	N/A	N/A	N/A	N/A	N/A
**PAC-1 binding**	**Result (% of positive platelets)**	**Normal range**	**PAC-1 binding**	**Result (% of positive platelets)**	**Normal range**	**PAC-1 binding**	**Result (% of positive platelets)**	**Normal range**	**PAC-1 binding**	**Result (% of positive platelets)**	**Normal range**
Resting	1.3	1.9-3.5	Resting	N/A	N/A	N/A	N/A	N/A	N/A	N/A	N/A
ADP 10 μM	59.2	53.9-76.04	ADP 10 μM	N/A	N/A	N/A	N/A	N/A	N/A	N/A	N/A
**Fibrinogen binding**	**Result (% of positive platelets)**	**Normal range**	**Fibrinogen binding**	**Result (% of positive platelets)**	**Normal range**	**Fibrinogen binding**	**Result (MFI)**	**Normal range (N≥75)**	**Fibrinogen binding**	**Result (MFI)**	**Normal range (N≥75)**
N/A	N/A	N/A	N/A	N/A	N/A	Resting	579	472 ± 138	Resting	383	472 ± 138
N/A	N/A	N/A	N/A	N/A	N/A	ADP 10 μM	1600	1494 ± 720	ADP 10 μM	7740	1494 ± 720
N/A	N/A	N/A	N/A	N/A	N/A	TRAP-6 25 μM	2371	2891 ± 1779	TRAP-6 25 μM	19951	2891 ± 1779
N/A	N/A	N/A	N/A	N/A	N/A	CRP 10 μg/ml	6586	9154 ±6984	N/A	N/A	9154 ±6984

Von Willebrand Factor (VWF)											
**VWF**	**Result (%)**	**Normal range**	**VWF**	**Result (%)**	**Normal range**	**VWF**	**Result (%)**	**Normal range**	**VWF**	**Result (%)**	**Normal range**
VWF:Ag	54	61-152%	VWF:Ag	57-65	61-152%	VWF:Ag	61	61-152%	VWF:Ag	119	61-152%
VWF:RCo	50	58-175%	VWF:RCo	48-61	58-175%	VWF:RCo	49	58-175%	VWF:RCo	114	58-175%

Genetic analysis			
CD36 (NM_001001548.3):c.660_669del (p.Asn220Lysfs*4)	*CD36* (NM_001001548.3):c.338_339del (p.Ser113Phefs*20)	*CD36*(NM_001001548.3):c.323_326del (p.Glu108AlafsTer48)	*CD36* (NM_001001548.3):c.649G>A (p.Gly217Arg)

Table S2. ACMG classification of the identified *CD36* variants.

Variant	ACMG criteria	ACMG classification
CD36(NM_001001548.3):c.660_669del	PVS1 (supports reduced expression), PM2 (supports bleeding), PP5 (supports reduced expression)	Likely Pathogenic
CD36(NM_001001548.3):c.338_339del	PVS1 (supports reduced expression), PM2 (supports bleeding)	Likely Pathogenic
CD36(NM_001001548.3):c.323_326del	PVS1 (supports reduced expression), PM2 (supports bleeding)	Likely Pathogenic
CD36(NM_001001548.3):c.649G>A	PP3 (supports reduced expression), PM2 (supports bleeding), PP5 (supports reduced expression), BP1	VUS
